# The Bath Additive KTPB Induces an Angiogenesis-Associated Transcriptional Program Without Detectable Cytotoxicity in Human Vascular Endothelial Cells

**DOI:** 10.3390/cimb48090895

**Published:** 2026-09-01

**Authors:** Norihiro Otani, Kieu D. M. Nguyen, Kiyoshi Maehara, Jiawei Wan, Atsushi Hirokawa, Takehito Sugasawa

**Affiliations:** 1 Doctoral Program in Sports Medicine, Graduate School of Comprehensive Human Sciences, University of Tsukuba, 1-1-1 Tennodai, Tsukuba 305-8572, Japan; 2 X-PLOSION LLC, 2F Yamazaki CA Building, 3-8-11 Ougicho, Naka-ku, Yokohama 231-0027, Japan; 3 Human Biology Program, University of Tsukuba, 1-1-1 Tennodai, Tsukuba 305-8572, Japan; 4 Doctoral Program in Medical Sciences, Graduate School of Comprehensive Human Sciences, University of Tsukuba, 1-1-1 Tennodai, Tsukuba 305-8572, Japan; 5 Laboratory of Sports Medicine, Department of Clinical Medicine, Institute of Medicine, University of Tsukuba, 1-1-1 Tennodai, Tsukuba 305-8572, Japan; 6 College of Medicine, School of Medicine and Health Sciences, University of Tsukuba, 1-1-1 Tennodai, Tsukuba 305-8572, Japan; 7 Center for Cyber Medicine Research, University of Tsukuba, 1-1-1 Tennodai, Tsukuba 305-8572, Japan

**Keywords:** bath additive, RNA sequencing, angiogenesis, wound healing, VEGFA-VEGFR2 signaling, cosmetic science

## Abstract

Regular bathing is a common habit worldwide, yet bath additives have been characterized almost exclusively at the physiological level, while their molecular effects on human cells remain largely unexplored. We previously showed by RNA sequencing (RNA-seq) that the complex bath additive Karada Totonou ProBath (KTPB) induced the expression of *EGR1* and hyaluronic acid synthase genes in human keratinocytes and fibroblasts, which represent the vascular endothelial growth factor (VEGF)-producing side of the cutaneous angiogenic axis; whether the VEGF-receiving endothelium responds to KTPB was unknown. Here, human vascular endothelial cells were exposed to KTPB and profiled by RNA-seq, and cytotoxicity was assessed using resazurin and Hoechst assays. KTPB altered gene expression in a time-dependent manner, and the differentially expressed genes were classified into three clusters with distinct temporal profiles: a late-repressed cluster, a transiently induced cell cycle-associated cluster, and a late-induced cluster enriched for blood vessel development and VEGFA-VEGFR2 signaling. The angiogenesis-related genes *ID1*, *ID3*, and *EDN1* were markedly upregulated, peaking at 1–2 h. KTPB showed no detectable cytotoxicity in this cell model at any of the tested concentrations, which spanned the recommended use range. These results indicate that KTPB elicits an angiogenesis-associated transcriptional program in vascular endothelial cells without compromising viability and highlight transcriptomics’ value for characterizing bath additives.

## 1. Introduction

Regular bathing is a habit deeply rooted in many cultures, and bath additives developed to enhance the physiological benefits of bathing have become a popular part of self-care routines in recent years. The global market for medicated bath additives has continued to expand steadily, with a value estimated at approximately USD 33.4 billion in 2025 and projected to reach USD 47.5 billion by 2030, corresponding to a compound annual growth rate of approximately 7%; this growth has been driven in part by the aging of the population and the increasing prevalence of degenerative joint and skin conditions [1].

Despite this widespread use of bath additives, little research has investigated their biological effects, especially their impact on gene expression. To address this research gap, we previously analyzed the effects of a newly developed complex bath additive, Karada Totonou ProBath (KTPB), on two human skin-derived cell lines (keratinocytes and fibroblasts) using RNA sequencing (RNA-seq) [2]. That study showed that KTPB induced significant changes in the expression of more than 80 genes in both cell lines, and that the genes encoding Early Growth Response Protein 1 (*EGR1*) and hyaluronic acid synthases (*HAS2* or *HAS3*) were upregulated over time. This suggested that KTPB may have effects related to angiogenesis and wound healing in skin cells.

Vascular endothelial cells also play a crucial role in the process of skin wound healing, and the angiogenesis that they drive is considered indispensable for successful tissue repair [3]. However, the effects of KTPB on these cells have not yet been investigated.

KTPB was originally developed as a bath additive for athletes. Athletes engaged in contact sports, such as basketball, combat sports, soccer, and rugby, frequently sustain skin injuries, including abrasions and lacerations, through friction and player-to-player or player-to-surface contact [4,5]. Such injuries disrupt the skin barrier and create open wounds. During wound healing, especially in the proliferative phase, angiogenesis driven by vascular endothelial cells plays an essential role in forming granulation tissue and supplying oxygen and nutrients to the regenerating tissue [6,7,8]. Because a disrupted skin barrier may allow bath water to reach the exposed wound bed, components of a bath additive dissolved in the bath water could plausibly come into contact with vascular endothelial cells when an athlete with such wounds takes a bath.

Although KTPB was originally developed for athletes, the burden of cutaneous injury is not confined to sport. For example, in cosmetic dermatology, controlled skin injury is deliberately induced: fractional photothermolysis creates microscopic columns of thermal injury, which heal through a wound-healing response that drives dermal remodeling [9,10]. The duration of the resulting interval of erythema, edema, and crusting, commonly termed downtime, is a principal determinant of patient satisfaction, and post-procedural erythema may persist and require additional intervention [11]. Because the proliferative phase of cutaneous repair depends on neovascularization, with angiogenic capillary sprouts invading the wound clot and organizing into a microvascular network that supplies granulation tissue [12], the competence of the endothelial compartment is directly relevant to how quickly treated skin regains its normal appearance. In this context, a bath additive could serve as an attractive delivery vehicle, carrying compounds that optimize wound healing to the cells themselves. Bathing is a non-invasive, whole-body form of care that requires no procedural expertise, and it can be repeated daily, and in some cases several times a day, throughout the recovery period. Characterizing how a bath additive acts on vascular endothelial cells is therefore not only a mechanistic question but also a question of cosmetic relevance, for athletes and general consumers alike.

In the present study, to investigate the effects of KTPB on vascular endothelial cells, we performed a time-course transcriptomic analysis of immortalized human umbilical vein endothelial cells (HUEhT-1) exposed to KTPB using RNA-seq and evaluated its cytotoxicity. We focused particularly on changes in the expression of angiogenesis-related genes and discussed the potential of KTPB to contribute to the wound healing process.

## 2. Materials and Methods

### 2.1. Cell Culture

Immortalized human umbilical vein endothelial cells (HUEhT-1; JCRB1458) were obtained from the JCRB Cell Bank (Osaka, Japan). The cells were cultured in Endothelial Cell Growth Medium 2 (Cat#C-22011; PromoCell, Heidelberg, Germany) on collagen-coated 10 cm dishes. The medium was supplemented with a penicillin–streptomycin mixed solution (Cat#09367-34; Nacalai Tesque, Kyoto, Japan) at 1% (*v*/*v*), giving final concentrations of 100 units/mL penicillin and 100 µg/mL streptomycin. The cells were maintained at 37 °C in a humidified atmosphere containing 5% CO_2_. The cells were detached by treatment with 0.025% trypsin–EDTA for passaging.

### 2.2. KTPB Preparation and Treatment

**Figure 1:**
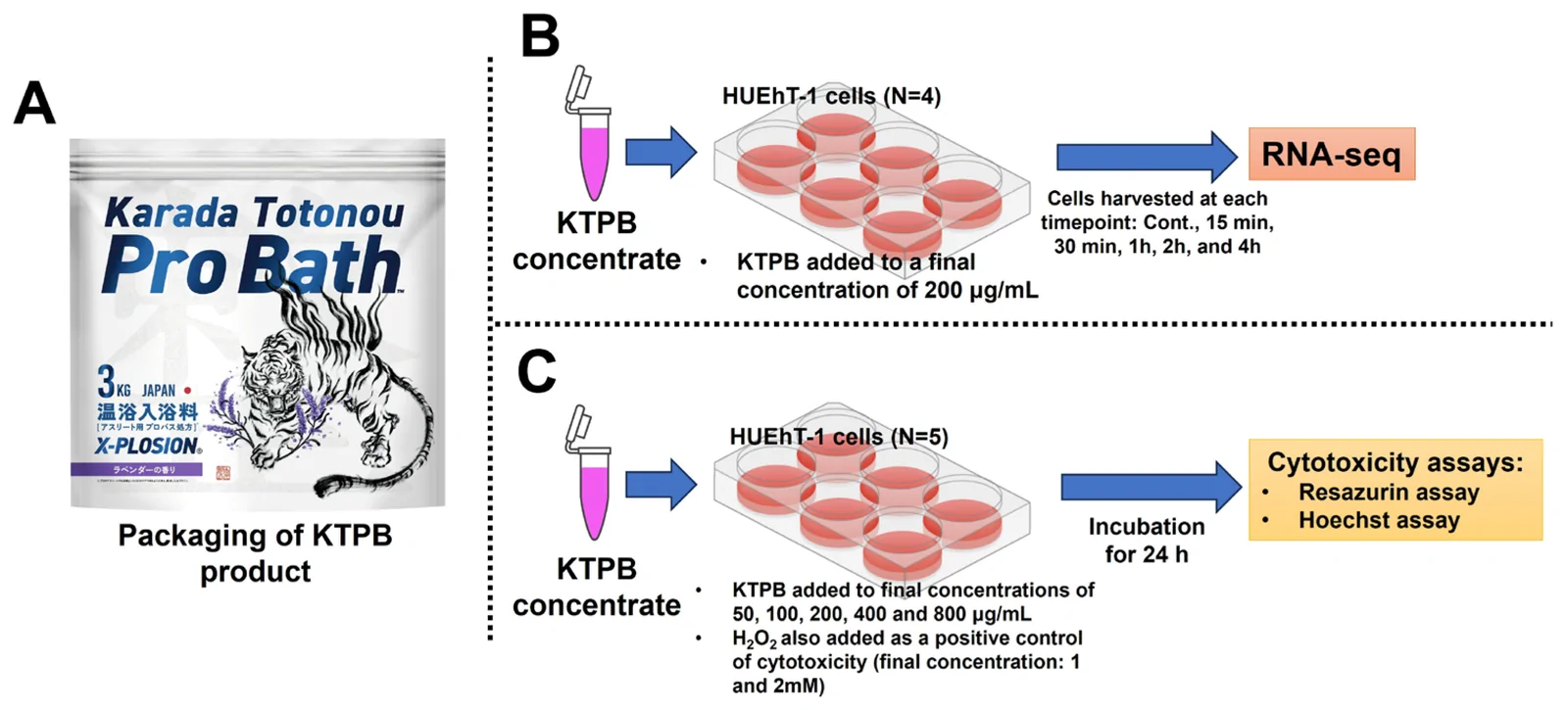
Overview of the experimental design. (**A**) Packaging of the bath additive Karada Totonou ProBath (KTPB), a powder-type product. (**B**) Experimental design for the RNA-seq analysis, in which HUEhT-1 cells were treated with KTPB and harvested at five timepoints after exposure, together with a control group (n = 4 per group). (**C**) Experimental design for the cytotoxicity assays, in which the cells were treated with KTPB or hydrogen peroxide (H_2_O_2_) as a positive control and evaluated using a resazurin assay and a Hoechst assay (n = 5 per group).

KTPB (Karada Totonou ProBath), a bath additive commercially available on the Japanese market (Figure 1A), is marketed by X-PLOSION LLC (Yokohama, Japan) and produced by a contract manufacturer on its behalf. The qualitative composition of KTPB, as declared by the manufacturer, is listed in Table 1. The exact quantitative formulation is proprietary and is not disclosed here. A single production lot (Lot#54180) was used for all experiments reported here; the product was purchased in March 2026 through ordinary retail channels and was therefore the same material as that available to consumers. Stock solutions were prepared immediately before each experiment: the powder was weighed and dissolved in Milli-Q water under aseptic conditions in a clean bench. For the RNA-seq experiment, a 20 mg/mL stock solution was used. The cells were first grown to confluence in six 10 cm collagen-coated dishes and then seeded at 1 × 10^5^ cells/well in collagen-coated six-well plates. The culture medium was replaced 24 h after seeding, and the cells were treated with KTPB a further 24 h later. The overall experimental design is shown in Figure 1. For the RNA-seq analysis, KTPB was added to the culture medium to a final concentration of 200 µg/mL (20.2 µL per well containing 2 mL of medium). For the control group (Con.), an equal volume of Milli-Q water—the vehicle used to prepare the KTPB stock solution—was added instead of KTPB. At 15 min, 30 min, 1 h, 2 h, and 4 h after KTPB addition, and at 2 h after Milli-Q water addition for the control group, the cells were lysed by directly applying 1 mL of QIAzol Lysis Reagent (Cat#79306; QIAGEN, Venlo, the Netherlands) to each well (n = 4 per group) (Figure 1B).

### 2.3. RNA Extraction, Library Preparation, and Sequencing

Total RNA was extracted and purified from the collected cells in our laboratory using the RNeasy Mini Kit (QIAGEN), in accordance with the manufacturer’s instructions. The assessment of RNA integrity, library preparation, and sequencing were performed by Novogene (Tokyo, Japan). The RNA integrity numbers (RINs) of all samples were ≥8.0, indicating that the RNA was intact and suitable for library preparation and sequencing. Poly(A) mRNA was isolated using the NEBNext Poly(A) mRNA Magnetic Isolation Module (Cat#E7490L; New England Biolabs, Ipswich, MA, USA), and sequencing libraries were prepared using the NEBNext Ultra II RNA Library Prep Kit for Illumina (Cat#E7770S; New England Biolabs), the same reagents as in our previous study [2]. Sequencing was performed on a NovaSeq X Plus System (Illumina, San Diego, CA, USA), generating approximately 3 Gb of data per sample, corresponding to approximately 20 million paired-end reads per sample.

### 2.4. Bioinformatic Analysis

The bioinformatic analysis was performed using CLC Genomics Workbench (version 26.0.2; QIAGEN). The obtained FASTQ files were mapped to the human reference genome (GRCh38) with the corresponding annotation file using the “RNA-Seq Analysis” tool. The mapping quality was confirmed using the report generated by the “RNA-Seq Analysis” tool. For all samples, the expected coverage bias was evaluated as “unbiased” in the transcript length coverage analysis, indicating that there was no bias toward the 3′ or 5′ end of the transcripts and that the reads were accurately mapped. In addition, the proportion of reads that were specifically mapped as pairs was approximately 97% for all samples. The gene expression levels were quantified as transcripts per million (TPM). TPM values were used for visualization, clustering, and the expression-level thresholds described below, whereas all statistical testing was performed on raw read counts. A principal component analysis (PCA) plot was generated using the “PCA for RNA-Seq” tool. Differential expression analysis was performed using the “Differential Expression for RNA-Seq” tool. This tool fits a negative binomial generalized linear model to the raw read counts of each gene (the “Total Exon Reads” values) and corrects for differences in library size among samples using the trimmed mean of M-values (TMM) normalization implemented within the tool. Two comparison modes of this tool were used. The “Across groups (ANOVA-like)” comparison, which tests for an overall group effect across all six groups and uses a likelihood ratio test, was used to extract the differentially expressed genes for clustering and for the Venn diagram analysis described below. The “All group pairs” comparison, which tests each pair of groups and uses a Wald test, was used for the analysis of the individual genes *ID1*, *ID3*, and *EDN1*. The FDR-corrected *p*-values from the “All group pairs” comparison were used to assess the significance of the expression changes at each timepoint relative to the control group for those individual genes. To extract the differentially expressed genes (DEGs) associated with KTPB treatment for the subsequent clustering, more stringent thresholds of a Bonferroni-corrected *p*-value < 0.01 and a maximum group mean >30 were applied. These thresholds were chosen for an exploratory time-course design with n = 4 per group: Bonferroni correction was used in place of the FDR in order to control false positives as stringently as possible, and the expression-level floor was applied to exclude genes whose apparent fold changes arise from very low read counts. The DEGs were subjected to hierarchical clustering and visualized as a heatmap using CLC Genomics Workbench, based on the Z-scores of their expression values. To identify genes with both high expression levels and large changes in expression, a Venn diagram analysis was performed using the web tool Venny 2.1 [13] based on three criteria: a Bonferroni-corrected *p*-value < 0.01, a maximum group mean >100, and a fold change >5. This higher expression floor and the fold-change criterion were applied only to this focused selection step, and not to the clustering analysis described above. The expression values and statistical results for all genes are provided in Table S1. The bar graphs and pie charts were created using Microsoft Excel (Office 2021; Microsoft, Redmond, WA, USA).

### 2.5. Gene Ontology and Pathway Enrichment Analyses

Gene Ontology (GO) and pathway enrichment analyses were performed for each gene cluster using Metascape [14]. The gene lists of the respective clusters were uploaded to Metascape, and the analysis was performed. The top 20 most enriched terms were extracted based on the corrected *p*-value (q-value) and were visualized as a bar graph ranked by −log10(P). To illustrate relationships among these terms, a network was constructed in which terms are represented as nodes connected by edges.

### 2.6. Quantitative Real-Time PCR

To confirm the RNA-seq results using an orthogonal measurement method, the expression levels of *ID1*, *ID3*, and *EDN1* were quantified by quantitative real-time PCR (qPCR). The method was similar to that used in our previous study [15], except for the points described below. Residual total RNA from the samples analyzed by RNA-seq was used (n = 4 per group). Accordingly, this qPCR analysis provides orthogonal technical validation using the same biological samples but does not constitute independent biological validation. cDNA was synthesized from 250 to 350 ng of total RNA using the PrimeScript RT Master Mix (Cat#RR036B; Takara Bio, Kusatsu, Japan), in accordance with the manufacturer’s instructions, and was then diluted 10-fold with Milli-Q water before use as the template. The primers for *ID1*, *ID3*, *EDN1*, and *HPRT* were designed using Primer-BLAST (version 2.5.0) [16], and their sequences are listed in Supplementary Table S2. qPCR was performed using PowerTrack SYBR Green Master Mix (Thermo Fisher Scientific, Waltham, MA, USA) with ROX as a reference dye, on a CronoSTAR 96 Real-Time PCR System (Takara Bio). The thermal cycling conditions were as follows: 50 °C for 2 min, 95 °C for 2 min, followed by 40 cycles of 95 °C for 5 s and 60 °C for 30 s. A melting curve analysis was then performed to confirm the specificity of the amplification. *HPRT* was used as the housekeeping gene, and the relative expression levels were calculated using the ΔΔCt method. All qPCR reactions were performed in duplicate.

### 2.7. Cytotoxicity Assays

The cytotoxicity of KTPB toward HUEhT-1 cells was evaluated using a resazurin assay and a Hoechst assay, which were performed sequentially on the same cells. The cells were seeded at 1 × 10^5^ cells/well in collagen-coated six-well plates and cultured as described above (medium replacement at 24 h, followed by treatment 24 h later). The cells were treated with KTPB at final concentrations of 50, 100, 200, 400, and 800 µg/mL for 24 h by adding 20.2 µL of KTPB stock solutions of 5, 10, 20, 40, and 80 mg/mL, respectively, to each well containing 2 mL of medium. For the control group (Con.), an equal volume (20.2 µL) of Milli-Q water was added instead of KTPB, as in the RNA-seq experiment. In addition, hydrogen peroxide (H_2_O_2_), used as a positive control for cytotoxicity, was added at final concentrations of 1 and 2 mM (n = 5 per group) (Figure 1C).

For the resazurin assay, a 1 mM resazurin stock solution was prepared by dissolving resazurin sodium salt (Cat#R0203; Tokyo Chemical Industry, Tokyo, Japan) in PBS, followed by filtration through a 0.22 µm filter. A resazurin-containing medium was prepared by adding the stock solution to the culture medium at 1/100 volume (e.g., 1 mL of the stock solution per 100 mL of medium) to give a final concentration of 10 µM. After the existing culture medium in each well was aspirated, the resazurin-containing medium was added, and the cells were incubated for 3 h. Subsequently, 100 µL of the culture supernatant from each well was dispensed in duplicate into a black 96-well plate, and the fluorescence intensity (excitation/emission wavelengths of 560/590 nm) was measured using a Varioskan LUX microplate reader (Thermo Fisher Scientific, Waltham, MA, USA). The measured fluorescence values were expressed as a percentage of the mean value of the control group (Con.), which was set to 100%.

For the Hoechst assay, the number of cells was estimated based on the DNA-binding fluorescence of Hoechst 33258, in accordance with the method of Rao and Otto [17]. After collecting the culture supernatant for the resazurin assay, the remaining supernatant was removed, and 1 mL of a solution of 1× saline-sodium citrate (SSC) containing 0.03% sodium dodecyl sulfate (SDS) was added to each well. The cells were lysed by pipetting. Then, 75 µL of each cell lysate was mixed with 75 µL of Cellstain Hoechst 33258 solution (Cat#H341; Dojindo Laboratories, Kumamoto, Japan; diluted 1:250 in 1× SSC) in a black 96-well plate in duplicate, and the fluorescence intensity was measured at excitation/emission wavelengths of 355/460 nm using a Varioskan LUX microplate reader (Thermo Fisher Scientific). The number of cells per well was determined from a standard curve, which showed a coefficient of determination (R^2^) of 0.99.

### 2.8. Statistical Analysis

The statistical analysis of the RNA-seq data is described in Section 2.4 (Bioinformatic Analysis). This section describes the statistical analysis of the other data, namely, those from the cytotoxicity assays and qPCR.

For the cytotoxicity assays, statistical analysis was performed using GraphPad Prism (version 11.0.0; GraphPad Software, Boston, MA, USA). The normality of the data was assessed using the Shapiro–Wilk test, followed by an evaluation of the homogeneity of variance using the Brown–Forsythe test. One-way ANOVA followed by the Tukey–Kramer post hoc test was then performed to compare the groups.

For the qPCR data, statistical analysis was also performed using GraphPad Prism. After confirming normality using the Shapiro–Wilk test, the homogeneity of variance was evaluated using the Brown–Forsythe test, and each timepoint was compared with the control group. For *ID3*, for which equal variance was assumed, Dunnett’s multiple comparison test was used; for *ID1* and *EDN1*, for which equal variance was not assumed, Dunnett’s T3 multiple comparison test was used.

A *p*-value < 0.05 was considered statistically significant.

## 3. Results

### 3.1. KTPB Alters the Gene Expression of Vascular Endothelial Cells in a Time-Dependent Manner

**Figure 2:**
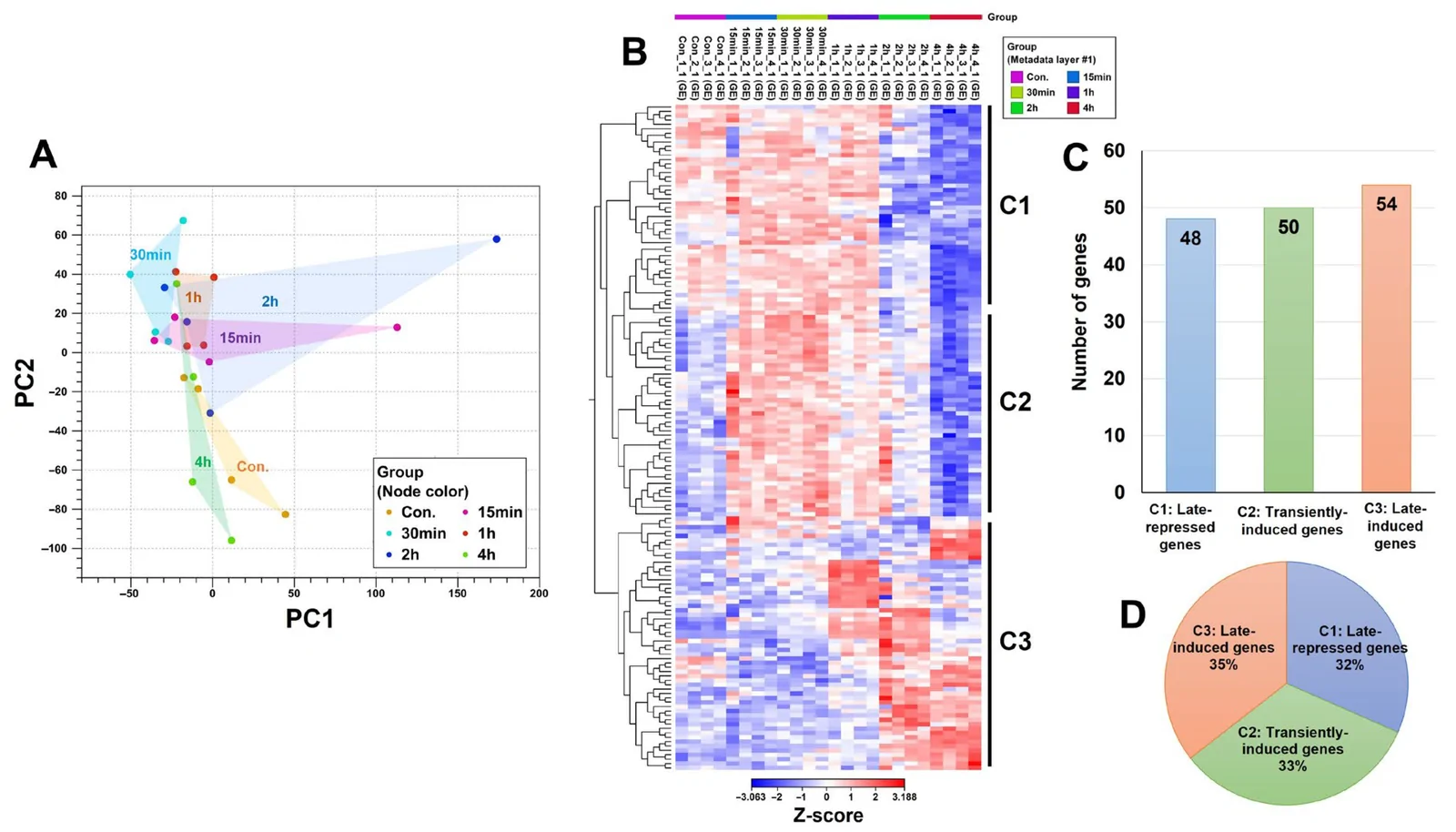
Transcriptomic changes in cultured vascular endothelial cells in response to KTPB exposure analyzed by RNA-seq. (**A**) Principal component analysis (PCA) plot of all samples (four biological replicates per group). Node colors indicate the experimental groups (Con., 15 min, 30 min, 1 h, 2 h, and 4 h). (**B**) Heatmap of the DEGs, visualized using Z-scores of the expression values. Hierarchical clustering classified the DEGs into three clusters (C1, C2, and C3). The color scale represents the Z-score, ranging from −3.063 (blue) to 3.188 (red). (**C**) Bar graph showing the number of genes in each cluster. (**D**) Pie chart showing the proportion of genes in each cluster. C1, late-repressed genes (n = 48 genes); C2, transiently induced genes (n = 50 genes); C3, late-induced genes (n = 54 genes).

To investigate the effects of KTPB on vascular endothelial cells, RNA-seq was performed on cells collected at five timepoints after KTPB exposure (15 min, 30 min, 1 h, 2 h, and 4 h) and on the control group, with four biological replicates per group. A principal component analysis (PCA) plot showed that, although the groups were not clearly separated from each other, the 30 min group was the most distinct from the control group along PC1 (Figure 2A). At the subsequent timepoints (1 h, 2 h, and 4 h), the plots gradually shifted back toward the position of the Con. group, indicating a transient change in the global gene expression profile that depended on the time elapsed since KTPB exposure.

When the genes with significant changes in expression were visualized in a heatmap, clear changes were observed depending on the time elapsed since the addition of KTPB (Figure 2B). A total of 152 DEGs were identified, and hierarchical clustering classified them into three clusters with distinct temporal expression profiles (C1, C2, and C3; Figure 2B). The genes in C1 (n = 48; 32%) showed decreased expression at the later timepoints and were therefore designated late-repressed genes. The genes in C2 (n = 50; 33%) showed a transient increase in expression at the intermediate timepoints and were designated transiently induced genes. The genes in C3 (n = 54; 35%) showed increased expression toward the later timepoints and were designated late-induced genes; this cluster included both genes that reached a peak at 1–2 h and genes that did so at 4 h (Figure 2C,D). These findings suggest that KTPB alters the gene expression of vascular endothelial cells in a time-dependent manner.

### 3.2. Gene Ontology and Pathway Enrichment Analyses of the Gene Clusters

**Figure 3:**
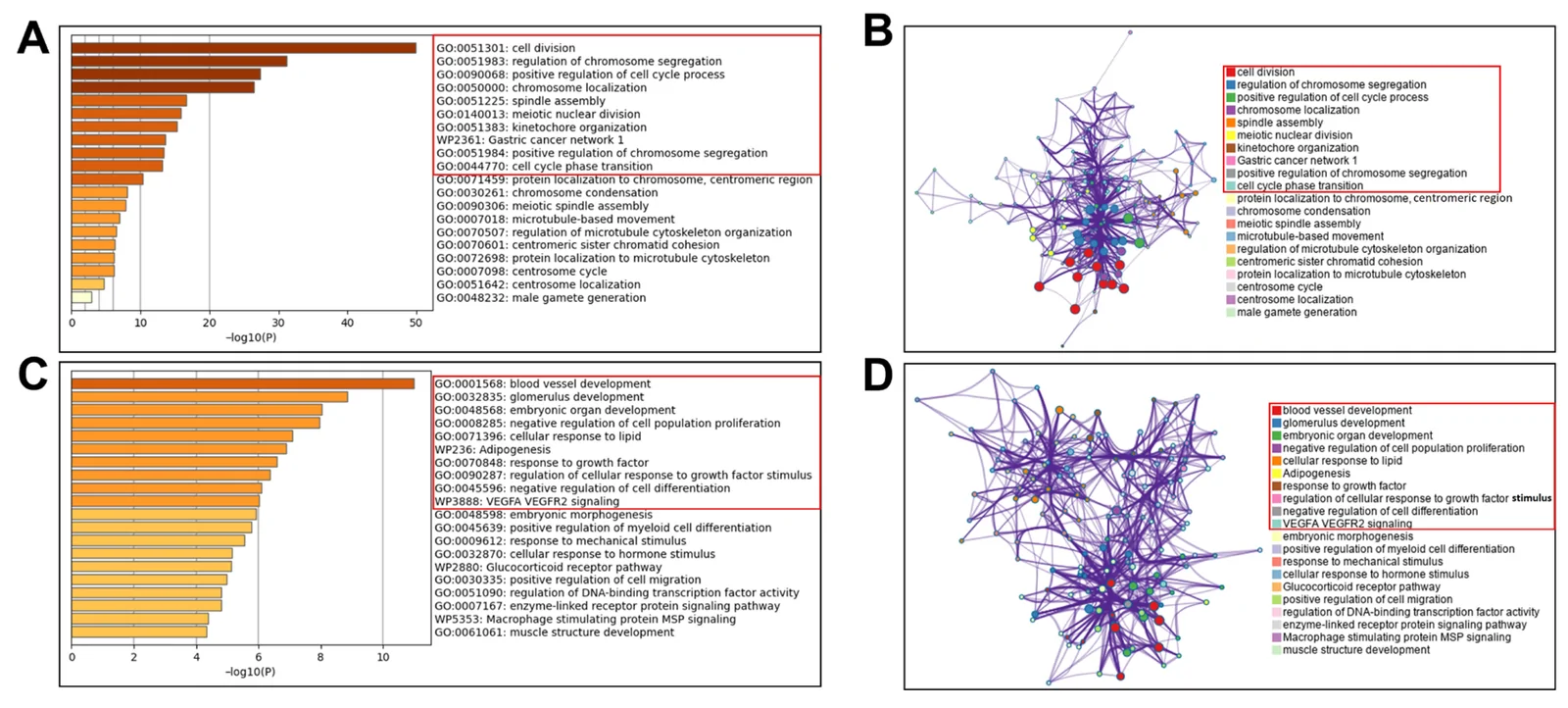
Gene Ontology (GO) and pathway enrichment analyses of the transiently induced and late-induced gene clusters (C2 and C3, respectively). Enrichment analysis was performed using Metascape. (**A**) Bar graph of the enriched terms for the C2 cluster (transiently induced genes), shown as −log10(P). (**B**) Network of the enriched terms for the C2 cluster, in which each node represents an enriched term and is colored according to its term identity, as shown in the legend. (**C**) Bar graph of the enriched terms for the C3 cluster (late-induced genes), shown as −log10(P). (**D**) Network of the enriched terms for the C3 cluster. In all panels, the red boxes indicate the top 10 most enriched terms.

To investigate the biological functions associated with the gene clusters identified by KTPB treatment, GO and pathway enrichment analyses were performed for each cluster using Metascape. For the transiently induced cluster (C2), the most enriched terms were predominantly related to cell division and the cell cycle, including “cell division,” “regulation of chromosome segregation,” and “positive regulation of cell cycle process” (Figure 3A,B). For the late-induced cluster (C3), the most significantly enriched term was “blood vessel development,” along with “response to growth factor,” “VEGFA-VEGFR2 signaling,” and “positive regulation of cell migration” (Figure 3C,D). In contrast, no significantly enriched terms were detected for the late-repressed cluster (C1).

These findings suggest that KTPB transiently alters the expression of genes related to cell division and the cell cycle and subsequently induces the expression of genes related to blood vessel development and angiogenesis in vascular endothelial cells.

### 3.3. KTPB Increases the Expression of ID1, ID3, and EDN1 in Vascular Endothelial Cells

**Figure 4:**
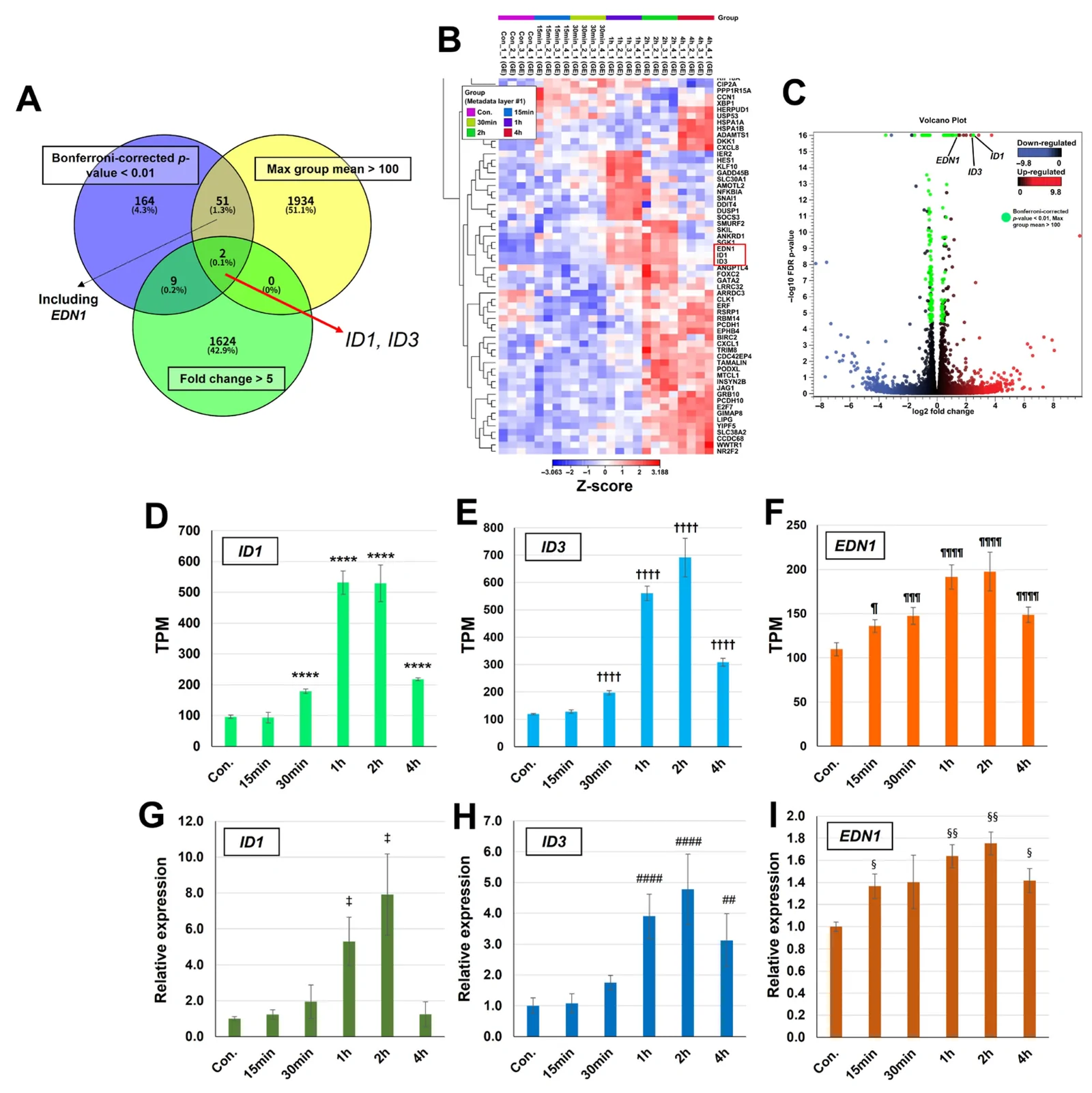
Identification of *ID1*, *ID3*, and *EDN1* as genes markedly upregulated by KTPB in vascular endothelial cells. (**A**) Venn diagram showing the number of genes satisfying each of the three criteria: a Bonferroni-corrected *p*-value < 0.01, a maximum group mean > 100, and a fold change > 5. *ID1* and *ID3* satisfied all three of these criteria, and *EDN1* satisfied the criteria for both the *p*-value and the fold change. (**B**) Heatmap indicating the positions of *EDN1*, *ID1*, and *ID3* (red box) among the differentially expressed genes. The color scale represents the Z-score, ranging from −3.063 (blue) to 3.188 (red). (**C**) Volcano plot of all genes, with *EDN1*, *ID1*, and *ID3* labeled. Red and blue dots indicate up- and downregulated genes, respectively. (**D**–**F**) Expression levels of *ID1* (**D**), *ID3* (**E**), and *EDN1* (**F**) as TPM values from the RNA-seq analysis at each timepoint. (**G**–**I**) Relative expression levels of *ID1* (**G**), *ID3* (**H**), and *EDN1* (**I**) determined by qPCR at each timepoint, normalized to the control group. All data are presented as the mean ± standard deviation (n = 4 per group). Symbols indicate the *p*-value versus the control group, with the number of symbols denoting the significance level (one, two, three, and four symbols for *p* < 0.05, *p* < 0.01, *p* < 0.001, and *p* < 0.0001, respectively): *, †, and ¶ for the TPM values of *ID1*, *ID3*, and *EDN1* (**D**–**F**); and ‡, #, and § for the corresponding qPCR values (**G**–**I**), respectively.

To identify genes with both high expression levels and large changes in expression in response to KTPB, a Venn diagram analysis was performed using three criteria: a Bonferroni-corrected *p*-value < 0.01, a maximum group mean > 100, and a fold change > 5 (Figure 4A). *ID1* and *ID3* satisfied all three of these criteria (Figure 4A). When these two genes were visualized using a volcano plot, both were confirmed to have large fold changes and small *p*-values (Figure 4C).

During the heatmap analysis, *EDN1* was found to be located immediately adjacent to *ID1* and *ID3*, showing a similar pattern of change in expression (Figure 4B). Because *EDN1* is a well-known gene related to angiogenesis, it was also selected for further analysis. *EDN1* satisfied the criteria for both the *p*-value and the fold change in the Venn diagram analysis (Figure 4A) and was confirmed to have a large fold change and a small *p*-value in the volcano plot (Figure 4C).

The expression of each gene was then quantified using the TPM value, and bar graphs were created (Figure 4D–F). After the addition of KTPB, the TPM values of *ID1*, *ID3*, and *EDN1* increased over time, reached a peak at 1–2 h, and then decreased at 4 h (Figure 4D–F). *ID1* and *ID3* reached their maxima at 1 h and 2 h, respectively, while *EDN1* reached its maximum at 2 h. To confirm these RNA-seq results by an orthogonal measurement method, the expression levels of the three genes were quantified by qPCR using the total RNA remaining from the same samples. The qPCR analysis reproduced the time-dependent upregulation of *ID1*, *ID3*, and *EDN1*, with peaks at 1–2 h (Figure 4G–I).

These findings suggest that KTPB increases the expression of *ID1*, *ID3*, and *EDN1* in vascular endothelial cells in a time-dependent manner.

### 3.4. KTPB Shows No Detectable Cytotoxicity Toward Vascular Endothelial Cells

To examine whether KTPB has any cytotoxic effect on vascular endothelial cells, HUEhT-1 cells were treated with it at final concentrations of 50–800 µg/mL for 24 h, and the cells were evaluated by phase-contrast microscopy, a resazurin assay, and a Hoechst assay. Hydrogen peroxide (H_2_O_2_) was used as a positive control for cytotoxicity.

**Figure 5:**
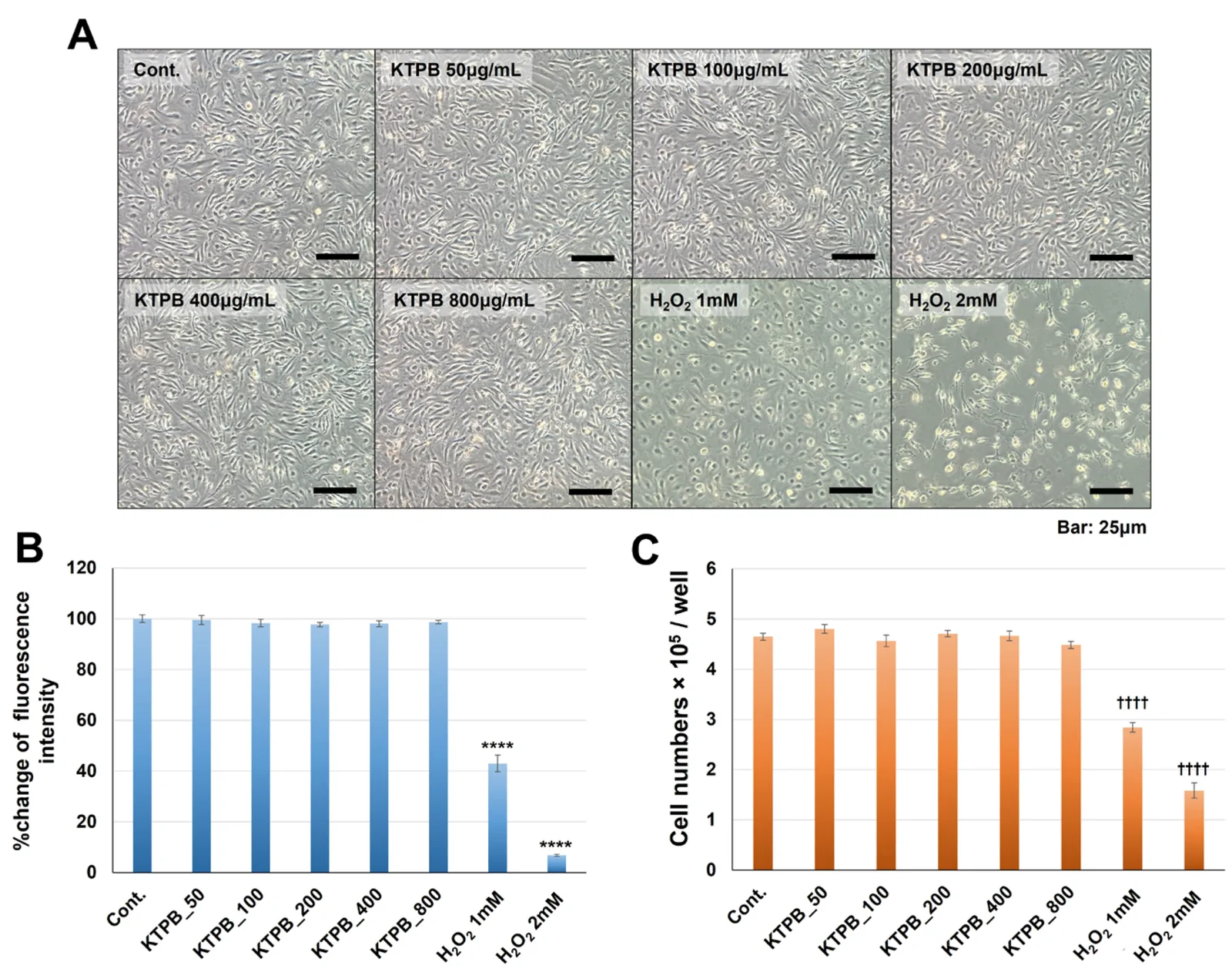
KTPB shows no detectable cytotoxicity toward HUEhT-1 cells. (**A**) Representative phase-contrast images of the cells in each group. Scale bars: 25 µm. (**B**) Cell viability evaluated using a resazurin assay, shown as a percentage of the fluorescence intensity of the control group. (**C**) Cell number evaluated using a Hoechst assay, shown as the number of cells per well (×10^5^). Data are presented as the mean ± standard deviation (n = 5 per group). **** and †††† indicate *p* < 0.0001 versus all other groups in (**B**) and (**C**), respectively.

In the phase-contrast images, the cells treated with KTPB at all tested concentrations retained a morphology comparable to that of the control group, whereas the cells treated with H_2_O_2_ showed clear morphological changes indicative of cell death, such as cell shrinkage and detachment (Figure 5A). Consistent with these observations, the resazurin assay showed that the fluorescence intensity was maintained at a level comparable to that of the control group across all KTPB concentrations, while it was significantly decreased by H_2_O_2_ in a dose-dependent manner (Figure 5B). Similarly, the Hoechst assay showed that the cell number was unchanged following KTPB treatment at all concentrations but was significantly decreased by H_2_O_2_ (Figure 5C).

These findings suggest that KTPB does not exhibit detectable cytotoxicity toward vascular endothelial cells at the concentrations used in this study, including the concentration used for the RNA-seq analysis (200 µg/mL).

## 4. Discussion

In this study, we focused on *ID1*, *ID3*, and *EDN1* as genes that were markedly upregulated by KTPB in vascular endothelial cells, with their expression reaching a peak at 1–2 h after exposure. These three genes are all closely related to the proliferation and angiogenic activity of endothelial cells; their coordinated upregulation therefore suggests that KTPB elicits a transcriptional response in endothelial cells that is associated with angiogenesis. Whether this translates into functional angiogenesis, or into the promotion of wound healing, was not tested here.

ID1 and ID3 are members of the inhibitor of DNA binding/differentiation (ID) family of helix-loop-helix proteins, which act as dominant-negative regulators of basic helix-loop-helix transcription factors and regulate angiogenesis. These two proteins play a critical role in promoting angiogenesis during development, tumor growth, and wound repair [18]. In endothelial cells, overexpression of *ID1* promotes their proliferation and angiogenesis in vitro [19], and *Id1* and *Id3* are required for the recruitment of bone marrow-derived endothelial cell precursors [20]. Furthermore, ID proteins can delay cellular differentiation and senescence and have been implicated in angiogenesis [21]. Their expression in endothelial cells is also upregulated by proangiogenic factors such as VEGF. In light of these previous reports, the increased expression of *ID1* and *ID3* following KTPB exposure suggests that KTPB may induce an angiogenesis- and proliferation-associated transcriptional program in vascular endothelial cells.

*EDN1* encodes endothelin-1 (ET-1), a vasoactive peptide that is secreted mainly by endothelial cells. In our analysis, *EDN1* was found to be located immediately adjacent to *ID1* and *ID3* in the heatmap and to show a similar pattern of change in expression, which prompted us to focus on this gene. In addition to its well-known role as a potent vasoconstrictor, ET-1 acts as a mitogen for endothelial cells and promotes their proliferation, migration, and invasion [22]. It has also been reported that ET-1 has a direct angiogenic effect on endothelial and perivascular cells, as well as an indirect effect through the increased release of VEGF [22,23]. The concurrent upregulation of *EDN1* with *ID1* and *ID3* therefore further supports the idea that KTPB induces an angiogenesis- and proliferation-associated transcriptional program in vascular endothelial cells.

It should be noted, however, that the role of ET-1 in wound healing is not necessarily straightforward. While the proangiogenic effects of ET-1 described above would be expected to support tissue repair, a contrasting report has shown that endothelial cell-specific ET-1 knockout mice exhibited accelerated cutaneous wound healing, accompanied by the downregulation of TGF-β, TNF-α, and connective tissue growth factor [24]. This apparent discrepancy should be interpreted in light of the fundamental differences between the two experimental systems. The accelerated wound healing was observed in mice in which ET-1 was constitutively absent from endothelial cells throughout development. Notably, the Tie2-Cre driver used in that model is active not only in endothelial cells but also in hematopoietic and myeloid lineages, including macrophages, which are themselves central effectors of wound healing [25,26]. In such a model, the chronic and complete loss of ET-1 is likely to elicit a cascade of secondary and higher-order compensatory changes in the surrounding cellular network, making it difficult to attribute the observed phenotype solely to the direct loss of endothelial ET-1. In contrast, the present study captured an acute and transient transcriptional response of endothelial cells, in which *EDN1* expression peaked at 1–2 h after KTPB exposure, reflecting the early and direct reaction of the cells before such compensatory mechanisms are established. Thus, the two findings are not necessarily contradictory but rather represent responses observed at different temporal and causal levels. Nevertheless, the functional consequences of the increase in *EDN1* expression observed here remain to be clarified, and further investigation will be required.

The GO and pathway enrichment analyses provided further insight into the cellular responses induced by KTPB. The late-induced cluster (C3), to which *ID1*, *ID3*, and *EDN1* belonged, was most significantly enriched for “blood vessel development” and also included “VEGFA-VEGFR2 signaling” and “response to growth factor.” In the enrichment network, these terms formed a loosely connected, multifaceted module centered on blood vessel development, together with terms related to growth factor responses, cell migration, and the regulation of differentiation (Figure 3D). This structure is consistent with the multi-step nature of angiogenesis, which requires the coordinated regulation of endothelial proliferation, migration, and differentiation. These results, together with the upregulation of *ID1*, *ID3*, and *EDN1*, support the idea that KTPB induces an angiogenesis-associated transcriptional program in vascular endothelial cells.

On the other hand, the transiently induced cluster (C2) was strongly enriched for terms related to cell division and the cell cycle. In contrast to the C3 network, the C2 enrichment network formed a single, densely connected module centered on “cell division” (Figure 3B), indicating that this cluster is functionally homogeneous and is driven by a coherent cell cycle-related program. While this might suggest that KTPB promotes the proliferation of endothelial cells, our Hoechst assay showed no significant increase in cell number following KTPB treatment (Figure 5C). This apparent inconsistency can be reconciled by considering the difference in the timescales of the two analyses: the RNA-seq analysis captured transient transcriptional changes within 4 h after KTPB exposure, in which the expression of the cell cycle-related genes of the C2 cluster peaked at 1–2 h, whereas the cell number was evaluated 24 h after exposure. Thus, the transient upregulation of cell cycle-related genes did not necessarily lead to a detectable increase in cell number under the present conditions.

Notably, the two clusters showed distinct temporal expression patterns: the cell cycle-related genes of the C2 cluster were upregulated transiently and peaked at the early-to-intermediate timepoints (1–2 h), whereas the angiogenesis-related genes of the C3 cluster, including *ID1*, *ID3*, and *EDN1*, were induced and remained elevated at the later timepoints. This temporal sequence—an early, transient induction of cell cycle-related genes followed by the later induction of angiogenesis-related genes—may reflect a coordinated transcriptional response in which KTPB first primes endothelial cells through cell cycle-related programs and subsequently drives an angiogenesis-associated transcriptional program. Such a stepwise process would be consistent with the proliferative phase of wound healing, in which endothelial proliferation and angiogenesis proceed in a temporally coordinated manner.

Taken together, the coordinated upregulation of *ID1*, *ID3*, and *EDN1*, along with the enrichment of angiogenesis-related terms, suggests that, in vascular endothelial cells, KTPB activates a transcriptional program associated with angiogenesis, which, if it were to translate into a functional response, could contribute to the wound healing process. Because angiogenesis in the proliferative phase plays an essential role in normal wound healing, in which vascular endothelial cells, fibroblasts, and keratinocytes proliferate in a coordinated manner to form granulation tissue, these findings point to a possible role for KTPB in supporting this process.

The present findings are highly consistent with and extend the findings of our previous study using cultured human skin cells (keratinocytes and fibroblasts) [2]. In that study, KTPB altered the expression of more than 80 genes and, importantly, the enrichment analysis revealed that genes related to angiogenesis (associated with VEGF signaling), cell migration, and the cell cycle were upregulated in both skin cell types. Expression of *VEGFA* was also increased in keratinocytes, and no detectable cytotoxicity was observed. The present study, conducted in vascular endothelial cells, recapitulated remarkably similar functional categories: the late-induced cluster was enriched for blood vessel development and VEGFA-VEGFR2 signaling, while the transiently induced cluster was enriched for the cell cycle, again without cytotoxicity. This convergence across three different cell types (keratinocytes, fibroblasts, and endothelial cells) suggests that the angiogenesis- and proliferation-associated transcriptional responses to KTPB are not cell-type-specific artifacts but rather reproducible features of the cellular response to KTPB.

Notably, the two studies are complementary with respect to the VEGFA-VEGFR2 axis. In the skin cells, KTPB upregulated the VEGF ligand (*VEGFA*), whereas in the present endothelial cells, the transcriptional response to KTPB was enriched for VEGFA-VEGFR2 signaling and included the upregulation of the angiogenesis-related genes *ID1*, *ID3*, and *EDN1* on the receiving side. During the proliferative phase of wound healing, angiogenesis proceeds through the paracrine and autocrine secretion of VEGF from keratinocytes, fibroblasts, and endothelial cells, which coordinately form granulation tissue. Our two studies therefore suggest that KTPB may act on both the VEGF-secreting cells (keratinocytes and fibroblasts) and the VEGF-responding cells (vascular endothelial cells), potentially supporting angiogenesis in a coordinated manner across the multiple cell types involved in cutaneous wound healing.

Because KTPB is a multi-component product, it is also informative to consider what is known about its individual ingredients (Table 1). Several of these have documented activity on vascular endothelial cells, and, notably, the reported effects do not all point in the same direction. An aqueous extract of *Citrus unshiu* peel—the same botanical material as that used in KTPB—has been reported to promote migration and capillary-like tube formation in human umbilical vein endothelial cells through phosphorylation of FAK and ERK1/2, with hesperidin and narirutin identified as major constituents that also showed proangiogenic effects [27]. An ethanolic extract of *Angelica dahurica*, a species of the same genus as the *Angelica acutiloba* used in KTPB, induces proliferation, migration, and tube formation in a human umbilical vein endothelial cell line through ERK1/2, Akt, and eNOS/NO signaling, and accelerates cutaneous wound healing in diabetic rats; those effects occur without any change in VEGF expression, indicating a direct action on the endothelium [28]. Conversely, capsaicin, the pungent principle of *Capsicum*, inhibits VEGF-induced endothelial proliferation, migration, and tube formation and causes G1 arrest through downregulation of cyclin D1 [29]; 6-gingerol likewise inhibits VEGF- and bFGF-induced endothelial proliferation and VEGF-induced tube formation [30].

These studies are not directly comparable with the present experiment. They examined purified compounds or single-herb extracts and assessed functional endothelial responses, including proliferation, migration, and tube formation, generally over longer exposure periods than in the present analysis, whereas the cells here were exposed to the whole product for up to 4 h and the readout was the transcriptional state of unstimulated endothelial cells. Taken together, this literature indicates that KTPB contains constituents with documented activity on vascular endothelial cells, acting through pathways that overlap with those enriched here, including ERK1/2 and VEGF-related signaling.

In terms of tolerability, KTPB showed no detectable cytotoxicity toward HUEhT-1 cells even at 800 µg/mL, the highest concentration tested. According to the manufacturer, the recommended amount of KTPB is 60–120 g per bath, which corresponds to a concentration of approximately 240–600 µg/mL when dissolved in a standard bathtub volume of 200–250 L. The concentration range tested in the present study (50–800 µg/mL) therefore fully covers this recommended range, and the absence of detectable cytotoxicity even at 800 µg/mL, which exceeds the upper end of the recommended concentration, indicates that KTPB is well tolerated by vascular endothelial cells under the conditions tested here. We emphasize that the absence of detectable cytotoxicity in a single immortalized endothelial cell line in vitro demonstrates cellular tolerability in this experimental model only; it does not establish the general safety of the product in humans, which would require appropriate in vivo and clinical evaluation.

These findings may have practical relevance for athletes, for whom KTPB was originally developed. The abrasions and lacerations common in contact sports disrupt the skin barrier, allowing bath water to reach the exposed wound bed. It is therefore plausible that KTPB components come into contact with cells in the wound environment, including vascular endothelial cells. We note that this is a hypothesis rather than an established exposure scenario: the local concentration of KTPB components actually reaching the dermal vasculature in vivo was not measured here and may differ substantially from the concentrations used in this study. Subject to that caveat, and given the angiogenesis-associated responses observed, KTPB may have the potential to support the healing of skin injuries in athletes, a possibility that remains to be tested.

Bath additives occupy an unusual position in cosmetic science. They are used regularly at scale, and their physiological effects on thermoregulation, peripheral circulation, and subjective recovery have been described for decades. However, related molecular findings have remained sparse. Unlike topical cosmetic ingredients, whose transcriptional effects on skin cells are routinely profiled, bath additives have almost never been examined at the level of gene expression, and, to our knowledge, our previous report [2] remains the only one describing a transcriptome-wide analysis of a bath additive’s effects on human cells. The present study is the direct continuation of that line of work, and it demonstrates why the approach is worth pursuing: neither the temporal structure of the response, a transient cell cycle cluster followed by a late angiogenesis-associated cluster, nor the specific induction of *ID1*, *ID3*, and *EDN1* would have been visible to the physiological assays that have historically defined this field. Transcriptomics does not merely add detail; it changes what questions can be asked about a bath additive, namely, whether it acts on the endothelium at all, on what timescale, and through which signaling axis.

From the perspective of cosmetic science, these findings generate a testable hypothesis. Because KTPB elicited an angiogenesis-associated transcriptional program in endothelial cells while showing no detectable cytotoxicity in this cell model across the entire recommended use range, it may be worth evaluating whether habitual bathing with KTPB supports the recovery of skin subjected to the controlled injury induced by esthetic procedures such as fractional laser resurfacing, chemical peeling, or microneedling, in which shortening downtime is a primary goal [10,11]. We emphasize that our data do not demonstrate such an effect; they establish only that the endothelial compartment is transcriptionally responsive to KTPB and that the additive is not cytotoxic at use-relevant concentrations. Two caveats deserve explicit mention. First, angiogenesis is context-dependent in the skin: whereas neovascularization is indispensable during the proliferative phase of repair, VEGF-driven angiogenesis has also been implicated in the pathogenesis of ultraviolet-induced photodamage and photoaging [31,32], so pro-angiogenic activity cannot be assumed to be beneficial in every cutaneous context. Second, sustained endothelial activation could in principle contribute to prolonged erythema. The transient kinetics observed here, with ID1, ID3, and EDN1 peaking at 1–2 h, argue against persistent activation, but this must be verified in vivo, ideally in human models of post-procedural repair.

This study has several limitations. First, the analysis was based on transcriptomic changes in cultured endothelial cells, and the expression of the corresponding proteins was not examined. Second, the actual angiogenic and wound healing responses, such as endothelial cell proliferation, migration, and tube formation, were not evaluated functionally. Third, KTPB is a complex mixture of multiple ingredients, and it remains undetermined which components are responsible for the observed changes in gene expression. The individual ingredients were not tested separately in this study, and the published literature on them does not resolve the question: as discussed above, KTPB contains constituents reported to promote endothelial angiogenic responses and others are reported to inhibit them. Synergistic or antagonistic interactions among the ingredients therefore cannot be excluded, and the net effect of the mixture cannot be predicted from studies of the isolated compounds. Future studies should test the individual ingredients and defined combinations of them at the proportions present in the product. Fourth, HUEhT-1 is an immortalized cell line derived from human umbilical vein endothelial cells and may not fully reproduce the biology of the dermal microvascular endothelial cells that mediate cutaneous wound healing; validation in primary human dermal microvascular endothelial cells would strengthen the translational relevance of these findings. Fifth, the design of the time course was not fully controlled. KTPB-treated cells were harvested at five timepoints, whereas the control group was harvested at a single timepoint (2 h) and used as a common reference; time-matched controls were not harvested alongside each treated group. Transcriptional changes intrinsic to the culture over time therefore cannot be formally separated from KTPB-associated changes, and the temporal profiles reported here should be interpreted with this in mind. Several features of the design constrain the likely magnitude of this confounding. The control group was harvested at the midpoint of the observation window rather than at time zero, so it is not systematically earlier or later in culture than the treated samples. The medium was not exchanged at the time of treatment. Instead, 20.2 µL of either KTPB stock solution or Milli-Q water was added directly to the 2 mL of medium in cultures that had been left undisturbed for the preceding 24 h. That addition corresponds to a 1.0% (*v*/*v*) dilution of the culture medium, and therefore to a reduction in osmolality of approximately 1%. In our previous study, in which twice this relative volume was added to cultures of human keratinocytes and fibroblasts, the osmolality of the medium was measured directly and was essentially unchanged relative to medium alone (332 mOsm/kg versus 339 mOsm/kg) [2]. For comparison, a reduction in osmolarity from 300 mOsm/L to 211 mOsm/L, a change of roughly 30%, has been shown to induce ERK1/2 phosphorylation and nuclear translocation in NIH-3T3 fibroblasts [33]. The observation window is also short relative to the growth kinetics of these cells: the doubling time of HUEhT-1 reported by the cell bank is approximately 37–58 h, and slower growth was observed during routine passaging under the conditions used here, so 4 h corresponds to approximately one-tenth or less of a single population doubling. Finally, the principal responses were non-monotonic in time, with separation from the control in the PCA being maximal at 30 min and the samples shifting back toward the control thereafter (Figure 2A), and with *ID1*, *ID3*, and *EDN1* peaking at 1–2 h and declining by 4 h (Figure 4D–I). For the monotonic components of the response, including cluster C1 and those genes within C3 that peaked at 4 h, a contribution of elapsed time in culture cannot be excluded. Time-matched controls should be included in future studies. Most importantly, because the present study was conducted using cultured cells in vitro, the results cannot be directly extrapolated to humans, and the potential of KTPB to promote wound healing should be regarded as a hypothesis to be tested. Future studies using protein-level analyses, functional angiogenesis assays, animal wound healing models, and ultimately verification in the actual wounds of athletes engaged in contact sports will be needed to confirm the physiological significance of these findings.

## 5. Conclusions

In this study, we used RNA-seq to investigate the effects of the bath additive KTPB on cultured human vascular endothelial cells. KTPB upregulated the angiogenesis-related genes *ID1*, *ID3*, and *EDN1* and induced the expression of genes related to blood vessel development and VEGFA-VEGFR2 signaling, with these transcriptional responses peaking at 1–2 h. No detectable cytotoxicity was observed across the recommended use range in this cell model. These changes define an angiogenesis-associated transcriptional program; functional angiogenesis was not assessed. Together with our earlier analyses of keratinocytes and fibroblasts, these findings place both the producing and the receiving sides of the cutaneous angiogenic axis within reach of a single bath additive. More broadly, transcriptomics adds a molecular dimension to a field long described only physiologically and offers a framework for evaluating future bath additives at the level of gene expression. In vivo validation remains necessary before any claim of clinical or cosmetic benefit can be made.

## Figures and Tables

**Table 1:** Ingredients of KTPB as declared by the manufacturer. The ingredients are grouped by function for readability; the quantitative formulation is proprietary and is not disclosed here. The same list has been reported previously [2].

Category	Ingredient
Inorganic salts	Sodium chloride, sodium sulfate, sodium bicarbonate
Amino acid salt	Sodium glutamate
Plant extracts	Unshu mikan (*Citrus unshiu*) peel extract, Angelica (*Angelica acutiloba*) root extract, Capsicum (*Capsicum frutescens*) fruit extract, ginger (*Zingiber officinale*) root extract, Cnidium (*Cnidium officinale*) rhizome extract
Enzymes	Protease, lipase
Excipients and carriers	Silica, cellulose gum, ethanol, water, butylene glycol
Colorants	Titanium dioxide, Blue No. 2 (CI 73015), Red No. 106 (Acid Red 52)

## Data Availability

The processed data underlying Figure 2, Figure 3, Figure 4 and Figure 5 are provided in the paper and the Supplementary Materials (Table S1: Expression values and statistical results for all genes). The raw NGS data (FASTQ, BAM, and other files) are not publicly available, as they are currently being used for secondary analyses to safeguard research priority; however, they may be shared upon reasonable request, provided that the intended use is clearly stated. KTPB is available free of charge from the authors for researchers wishing to replicate the experiments and is also commercially available from various websites in Japan. Further inquiries can be directed to the corresponding author.

## Supplementary Materials

The following supporting information can be downloaded at: https://www.mdpi.com/article/10.3390/cimb48090895/s1.

## Abbreviations

The following abbreviations are used in this manuscript:

ANOVA	analysis of variance
bFGF	basic fibroblast growth factor
cDNA	complementary DNA
DEG	differentially expressed gene
EDN1	endothelin-1 gene
EGR1	early growth response protein 1
eNOS	endothelial nitric oxide synthase
ERK	extracellular signal-regulated kinase
ET-1	endothelin-1
FAK	focal adhesion kinase
FDR	false discovery rate
GO	Gene Ontology
HAS	hyaluronic acid synthase
HUEhT-1	immortalized human umbilical vein endothelial cell line
ID	inhibitor of DNA binding/differentiation
KTPB	Karada Totonou ProBath
NO	nitric oxide
PBS	phosphate-buffered saline
PCA	principal component analysis
qPCR	quantitative real-time polymerase chain reaction
RIN	RNA integrity number
RNA-seq	RNA sequencing
SDS	sodium dodecyl sulfate
SSC	saline-sodium citrate
TGF-β	transforming growth factor-β
TMM	trimmed mean of M-values
TNF-α	tumor necrosis factor-α
TPM	transcripts per million
VEGF	vascular endothelial growth factor
VEGFR2	vascular endothelial growth factor receptor 2
